# Supplementary material for: Demonstration of targeted crossovers in hybrid maize using CRISPR technology
Source: Commun Biol. 2022 Jan 13;5:53. doi: 10.1038/s42003-022-03004-9 (PMC8758740; doi:10.1038/s42003-022-03004-9)
Supplement: Supplementary file 2 — Supplementary Information [file 42003_2022_3004_MOESM2_ESM.pdf]

Supplementary Table 1: Total number of BC1-F1 seeds analyzed by genotyping in the first genome editing experiment using gRNA1 grouped by reciprocal backcrosses

Construct	Back-Cross Type	Events # *	Total Seeds Genotyped
Editing: pCpf1_gRNA1	F1-T0(female) by Parent B(male)	26	2625 (62.5%)
Editing: pCpf1_gRNA1	Parent B(female) by F1-T0 (male)	21	1575 (37.5%)
Control: pCpf1_gRNA_control	F1-T0(female) by Parent B(male)	8	1015 (45%)
Control: pCpf1_gRNA_control	Parent B(female) by F1-T0 (male)	10	1250 (55%)

* BC1 seeds from both reciprocal backcrosses were genotyped for some F1-T0 events; some F1-T0 events were represented by only one type of the backcross

Supplementary Table 2: Genotyped BC1-F1 seeds grouped by reciprocal backcrosses

BC1-F1	F1-T0 (female) by Parent B (male)	Parent B (female) by F1-T0 (male)	Total Seeds Genotyped
Event1	308	0	308
Event2	270	41	311
Event3	329	132	461

Supplementary Table 3: The Chi-square contingency table used to validate statistical significance of the observed difference in recombination frequency between the control and treatment samples

	Seeds with No Cross-over	Seeds with Cross-over	Total
Treatment	4170	30	4200
Control	2264	1	2265
Total	6434	31	6465

Supplementary Figure 1: The guided nuclease editing activity in the F1-T0 plants.

a.

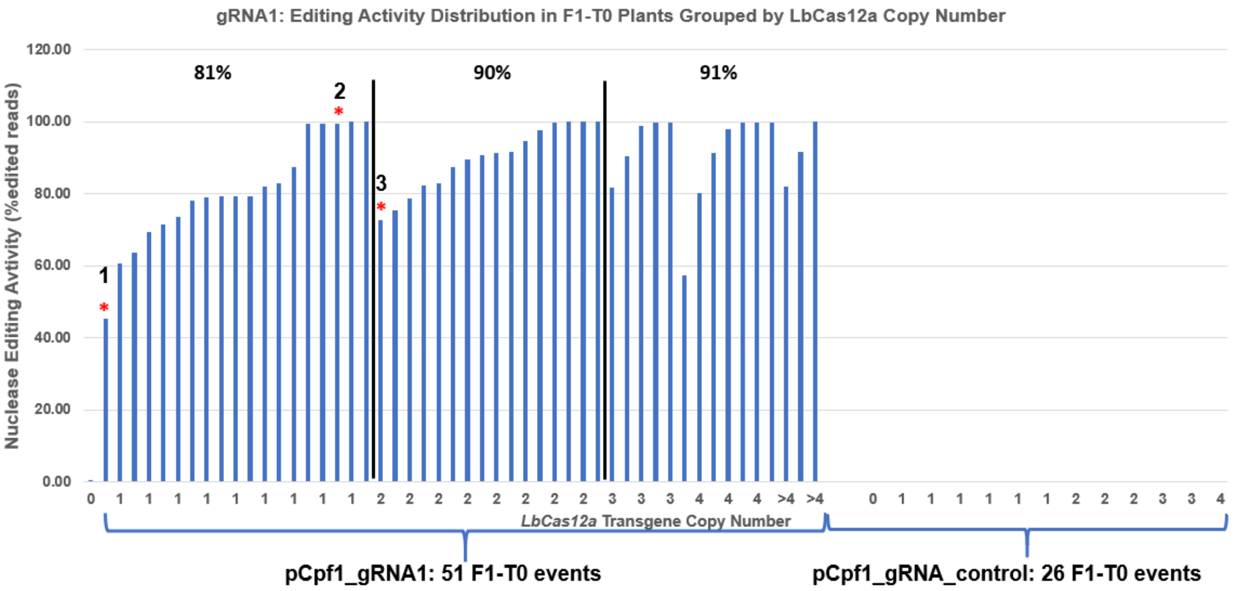

b.

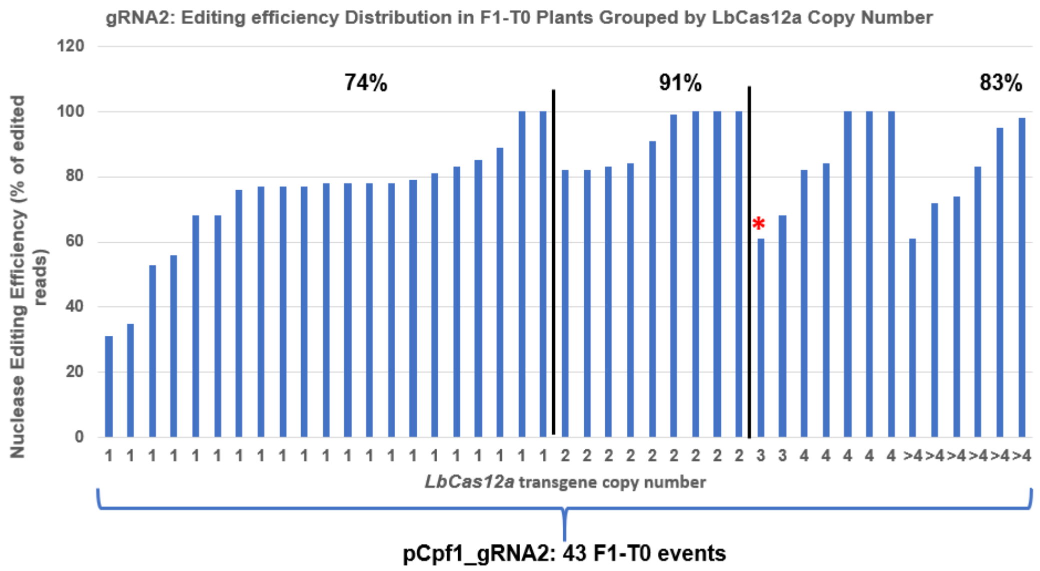

a. gRNA1 (pCpf1_gRNA1) or control (pCpf1_gRNA_control) plasmids. **b.** gRNA2 (pCpf1_gRNA2). DNA was isolated from a single leaf disc sampled from each F1-T0 plant for library construction and sequencing. Each bar on the graph represents a result derived from a single BC-F1 plant (n=1). The graphs show the distribution of editing activity grouped by T-DNA copy number. The numbers above the bar plot represent the average editing activity in events with T-DNA copy numbers from of 1, 2, or ≥3. Red stars indicate F1-T0 plants where the targeted CO was shown. Number 1, 2, and 3 in Figure1a represent the F1-T0 Events #1, #2, and #3, respectively.

Supplementary Figure 2: Examples of the DNA editing patterns in somatic cells.

a.

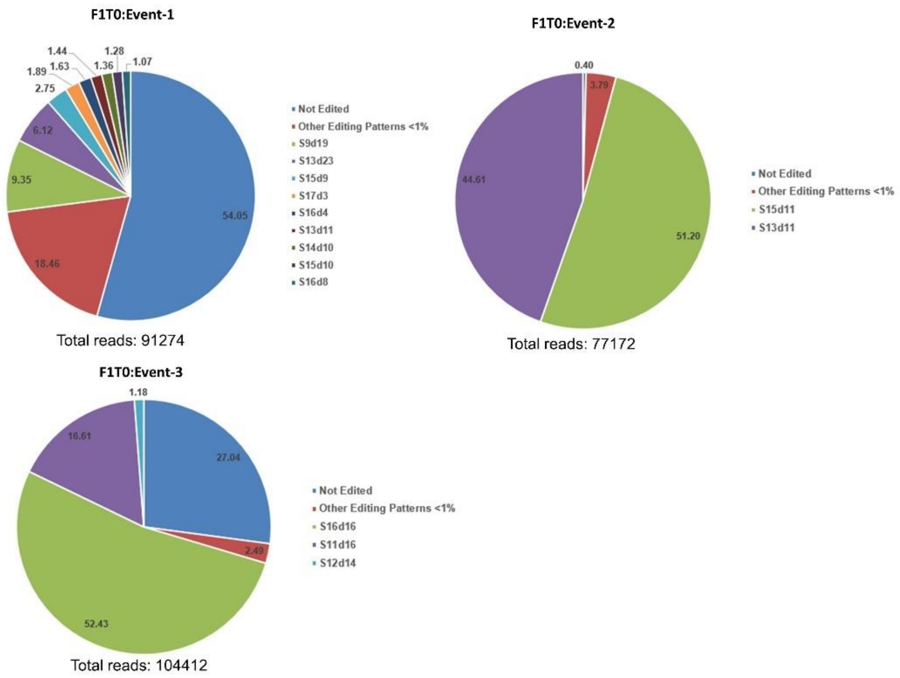

b.

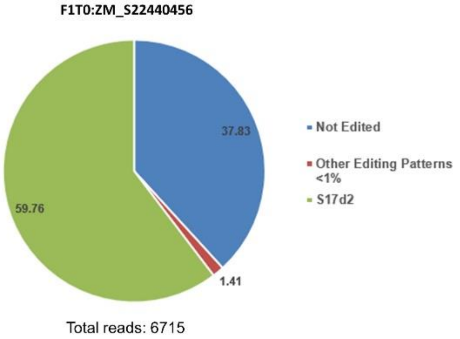

The four F1-T0 plants in which the targeted CO was identified in the backcrossed progeny are shown. **a.** Distribution of the DNA editing patterns in the F1-T0 plants transformed with

pCpf1_gRNA1 plasmid including Event #1, Envent#2, and Event#3 **b.** Distribution of the DNA editing patterns in the F1-T0 plant: ZM_S22440456 transformed with pCpf1_gRNA2 plasmid. An abbreviated description of the DNA editing patterns is presented as S#d#. **S#** indicates the nucleotide position in 23nt gRNA target sequence where the deletion starts; d# indicates the length of the deleted nucleotides. Only editing patterns >= 1% in the sequencing library are shown on the charts. The pie chart section: "Other Editing Patterns < 1%" represents combined proportions of all other editing patterns which are below 1% in the sequencing library.

Supplementary Figure 3: The marker genotypes of the BC1-F1 seeds with the targeted CO identified in the genome editing experiments with gRNA1 and gRNA2, respectively.

a.

Recomb#	Event	Construct	M1	M2	M3	M4	M5	M6	M7	M8	gRNA1	M9	M10	M11	M12	M13	M14	M15	M16	M17	M33	Cpf1	Recomb.Genotype
1	Event 1.1	Edited event	TT	GG	GG	CC	TT	CC	AA	AA		AG	GC	CT	TC	TG	GA	TC	AG	GA	TC	POS	B/A
2	Event 1.2	Edited event	TT	GG	GG	CC	TT	CC	AA	AA		AG	GC	CT	TC	TG	GA	TC	AG	GA	TC	POS	B/A
3	Event 1.3	Edited event	TT	GG	GG	CC	TT	CC	AA	AA		AG	GC	CT	TC	TG	GA	TC	AG	GA	TC	POS	B/A
4	Event 1.4	Edited event	TT	GG	GG	CC	TT	CC	AA	AA		AG	GC	CT	TC	TG	GA	TC	AG	GA	TC	NEG	B/A
5	Event 1.5	Edited event	TT	GG	GG	CC	TT	CC	AA	AA		AG	GC	CT	TC	TG	GA	TC	AG	GA	TC	NEG	B/A
6	Event 1.6	Edited event	TT	GG	GG	CC	TT	CC	AA	AA		AG	GC	CT	TC	TG	GA	TC	AG	GA	TC	POS	B/A
7	Event 2.1	Edited event	TT	GG	GG	CC	TT	CC	AA	AA		AG	GC	CT	TC	TG	GA	TC	AG	GA	TC	POS	B/A
8	Event 2.2	Edited event	TT	GG	GG	CC	TT	CC	AA	AA		AG	GC	CT	TC	TG	GA	TC	AG	GA	TC	NEG	B/A
9	Event 2.3	Edited event	TT	GG	GG	CC	TT	CC	AA	AA		AG	GC	CT	TC	TG	GA	TC	AG	GA	TC	POS	B/A
10	Event 2.4	Edited event	TT	GG	GG	CC	TT	CC	AA	AA		AG	GC	CT	TC	TG	GA	TC	AG	GA	TC	POS	B/A
11	Event 4.1	Control event	TT	GG	GG	CC	TT	CC	AA	AA		AG	GC	CT	TC	TG	GA	TC	AG	GA	TC	POS	B/A
12	Event 1.7	Edited event	CT	AG	AG	TC	AT	GC	TA	GA		GG	CC	TT	CC	GG	AA	CC	GG	AA	CC	NEG	A/B
13	Event 1.8	Edited event	CT	AG	AG	TC	AT	GC	TA	GA		GG	CC	TT	CC	GG	AA	CC	GG	AA	CC	NEG	A/B
14	Event 1.9	Edited event	CT	AG	AG	TC	AT	GC	TA	GA		GG	CC	TT	CC	GG	AA	CC	GG	AA	CC	NEG	A/B
15	Event 1.10	Edited event	CT	AG	AG	TC	AT	GC	TA	GA		GG	CC	TT	CC	GG	AA	CC	GG	AA	CC	POS	A/B
16	Event 1.11	Edited event	CT	AG	AG	TC	AT	GC	TA	GA		GG	CC	TT	CC	GG	AA	CC	GG	AA	CC	NEG	A/B
17	Event 1.12	Edited event	CT	AG	AG	TC	AT	GC	TA	GA		GG	CC	TT	CC	GG	AA	CC	GG	AA	CC	POS	A/B
18	Event 1.13	Edited event	CT	AG	AG	TC	AT	GC	TA	GA		GG	CC	TT	CC	GG	AA	CC	GG	AA	CC	POS	A/B
19	Event 1.14	Edited event	CT	AG	AG	TC	AT	GC	TA	GA		GG	CC	TT	CC	GG	AA	CC	GG	AA	CC	NEG	A/B
20	Event 1.15	Edited event	CT	AG	AG	TC	AT	GC	TA	GA		GG	CC	TT	CC	GG	AA	CC	GG	AA	CC	POS	A/B
21	Event 1.16	Edited event	CT	AG	AG	TC	AT	GC	TA	GA		GG	CC	TT	CC	GG	AA	CC	GG	AA	CC	POS	A/B
22	Event 1.17	Edited event	CT	AG	AG	TC	AT	GC	TA	GA		GG	CC	TT	CC	GG	AA	CC	GG	AA	CC	POS	A/B
23	Event 1.18	Edited event	CT	AG	AG	TC	AT	GC	TA	GA		GG	CC	TT	CC	GG	AA	CC	GG	AA	CC	NEG	A/B
24	Event 2.5	Edited event	CT	AG	AG	TC	AT	GC	TA	GA		GG	CC	TT	CC	GG	AA	CC	GG	AA	CC	POS	A/B
25	Event 2.6	Edited event	CT	AG	AG	TC	AT	GC	TA	GA		GG	CC	TT	CC	GG	AA	CC	GG	AA	CC	NEG	A/B
26	Event 2.7	Edited event	CT	AG	AG	TC	AT	GC	TA	GA		GG	CC	TT	CC	GG	AA	CC	GG	AA	CC	POS	A/B
27	Event 2.8	Edited event	CT	AG	AG	TC	AT	GC	TA	GA		GG	CC	TT	CC	GG	AA	CC	GG	AA	CC	POS	A/B
28	Event 2.9	Edited event	CT	AG	AG	TC	AT	GC	TA	GA		GG	CC	TT	CC	GG	AA	CC	GG	AA	CC	POS	A/B
29	Event 2.10	Edited event	CT	AG	AG	TC	AT	GC	TA	GA		GG	CC	TT	CC	GG	AA	CC	GG	AA	CC	NEG	A/B
30	Event 2.11	Edited event	CT	AG	AG	TC	AT	GC	TA	GA		GG	CC	TT	CC	GG	AA	CC	GG	AA	CC	NEG	A/B
31	Event 3.1	Edited event	CT	AG	AG	TC	AT	GC	TA	GA		GG	CC	TT	CC	GG	AA	CC	GG	AA	CC	NEG	A/B

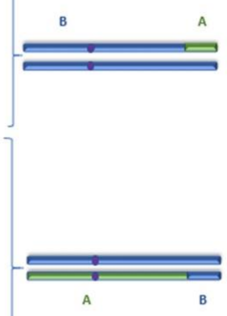

b.

Recomb#	Event	Construct	M3	M16	M17	M18	M19	M20	M21	M22	M23	M24	M25	gRNA2	M26	M27	M28	M29	M30	M31	M32	M33	M34	Cpf1	Recomb.genotype
1	ZM_S22440456	Editing	AG	AG	AG	AG	AG	AG	AG	AG	CT	AG	GT		CC	AA	AA	TT	AA	AA	GG	TT	GG	POS	B/A
2	ZM_S22440456	Editing	AG	AG	AG	AG	AG	AG	AG	AG	CT	AG	GT		CC	AA	AA	TT	AA	AA	GG	TT	GG	NEG	B/A
3	ZM_S22440456	Editing	AG	AG	AG	AG	AG	AG	AG	AG	CT	AG	GT		CC	AA	AA	TT	AA	AA	GG	TT	GG	NEG	B/A
4	ZM_S22440456	Editing	AG	AG	AG	AG	AG	AG	AG	AG	CT	AG	GT		CC	AA	AA	TT	AA	AA	GG	TT	GG	POS	B/A
5	ZM_S22440456	Editing	AG	AG	AG	AG	AG	AG	AG	AG	CT	AG	GT		CC	AA	AA	TT	AA	AA	GG	TT	GG	NEG	B/A
6	ZM_S22440456	Editing	AG	AG	AG	AG	AG	AG	AG	AG	CT	AG	GT		CC	AA	AA	TT	AA	AA	GG	TT	GG	NEG	B/A
7	ZM_S22440456	Editing	AG	AG	AG	AG	AG	AG	AG	AG	CT	AG	GT		CC	AA	AA	TT	AA	AA	GG	TT	GG	NEG	B/A
8	ZM_S22440456	Editing	AG	AG	AG	AG	AG	AG	AG	AG	CT	AG	GT		CC	AA	AA	TT	AA	AA	GG	TT	GG	NEG	B/A
9	ZM_S22440456	Editing	AG	AG	AG	AG	AG	AG	AG	AG	CT	AG	GT		CC	AA	AA	TT	AA	AA	GG	TT	GG	POS	B/A
10	ZM_S22440456	Editing	AG	AG	AG	AG	AG	AG	AG	AG	CT	AG	GT		CC	AA	AA	TT	AA	AA	GG	TT	GG	NEG	B/A
11	ZM_S22440456	Editing	AA	AA	GG	AA	GG	GG	GG	GG	CC	GG	TT		CG	AG	AT	GT	AG	AG	GT	CT	AG	NEG	A/B
12	ZM_S22440456	Editing	AA	AA	GG	AA	GG	GG	GG	GG	CC	GG	TT		CG	AG	AT	GT	AG	AG	GT	CT	AG	NEG	A/B
13	ZM_S22440456	Editing	AA	AA	GG	AA	GG	GG	GG	GG	CC	GG	TT		CG	AG	AT	GT	AG	AG	GT	CT	AG	POS	A/B
14	ZM_S22440456	Editing	AA	AA	GG	AA	GG	GG	GG	GG	CC	GG	TT		CG	AG	AT	GT	AG	AG	GT	CT	AG	NEG	A/B
15	ZM_S22440456	Editing	AA	AA	GG	AA	GG	GG	GG	GG	CC	GG	TT		CG	AG	AT	GT	AG	AG	GT	CT	AG	NEG	A/B
16	ZM_S22440456	Editing	AA	AA	GG	AA	GG	GG	GG	GG	CC	GG	TT		CG	AG	AT	GT	AG	AG	GT	CT	AG	POS	A/B
17	ZM_S22440456	Editing	AA	AA	GG	AA	GG	GG	GG	GG	CC	GG	TT		CG	AG	AT	GT	AG	AG	GT	CT	AG	POS	A/B
18	ZM_S22440456	Editing	AA	AA	GG	AA	GG	GG	GG	GG	CC	GG	TT		CG	AG	AT	GT	AG	AG	GT	CT	AG	NEG	A/B
19	ZM_S22440456	Editing	AA	AA	GG	AA	GG	GG	GG	GG	CC	GG	TT		CG	AG	AT	GT	AG	AG	GT	CT	AG	NEG	A/B
20	ZM_S22440456	Editing	AA	AA	GG	AA	GG	GG	GG	GG	CC	GG	TT		CG	AG	AT	GT	AG	AG	GT	CT	AG	NEG	A/B

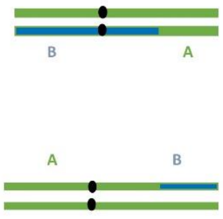

a: The marker genotypes for all BC1-F1 seeds with the targeted CO identified in the first genome editing experiment using gRNA1. **b**: The marker genotypes of the BC1-F1 recombinant seeds identified in the second genome editing experiment using gRNA2 (a subset of 10 examples

for each reciprocal cross direction is shown, out of the total 175 BC1-F1 targeted CO plants identified from this T0 event).
