# Supplementary material for: Demonstration of targeted crossovers in hybrid maize using CRISPR technology
Source: Commun Biol. 2022 Jan 13;5:53. doi: 10.1038/s42003-022-03004-9 (PMC8758740; doi:10.1038/s42003-022-03004-9)
Supplement: Supplementary file 3 — Description of Additional Supplementary Files [file 42003_2022_3004_MOESM3_ESM.pdf]

Description of Additional Supplementary Files

File name: Supplementary Data 1
Description: Source data underlying all graphs and charts in the main manuscript.

File name: Supplementary Data 2
Description: Primers sequences used in the study.

File name: Supplementary Data 3
Description: Genomic positions od SNPs assayed in the study.

File name: Supplementary Data 4
Description: Genotyping analysis of the BC1-F2 segregating population.

File name: Supplementary Data 5
Description: DNA sequences of the plasmid elements including promoters and genes.
